# Subclinical cardiovascular abnormalities in patients with juvenile systemic lupus erythematosus

**DOI:** 10.1186/1546-0096-9-S1-O20

**Published:** 2011-09-14

**Authors:** Nur Canpolat, Ozgur Kasapcopur, Salim Caliskan, Selman Gokalp, Meltem Bor, Mehmet Tasdemir, Lale Sever, Nil Arisoy

## Background and aim

Systemic lupus erythematosus (SLE) have accelerated atherosclerosis and increased risk of cardiovascular disease. It is thought that the atherosclerosis process begins in the first decades of life and progresses silently. The aim of this study is to evaluate subclinical cardiovascular abnormalities including atherosclerosis and left ventricular hypertrophy (LVH) in children and adolescent with SLE.

## Methods

Non-invasive cardiovascular assessments were made on 22 patients with juvenile SLE (20 girls, aged 15.0 ± 2.7 years) with GFR ≥90 ml/min/1.73 m^2^, following in a single center. Ambulatory blood pressure monitoring (ABPM) was used for the definition of hypertension. Ultrasonographic examinations of the heart and carotid artery were performed to evaluate LVH, carotid intima-media thickness (IMT) and carotid artery distensibility. Aortic (carotid-femoral) pulse wave velocity (PWV) and pulse wave analysis (PWA) were also performed for the assessment of arterial stiffness. To compare the PWV and PWA of the patients, two control groups were formed; matched for age/sex (control A; n=22) and for height/sex (control H; n=22).

## Results

The median disease duration was 39.5 (iqr 26.0-65.2) months. Twenty patients fulfilled four or more of the SLE ACR criteria for SLE classification; 2 patients had 3 criteria. Twelve patients had active disease characterized by SLEDAI ≥3 and none of the patients had organ damage as identified by SLICC. Eight patients (36%) had hypertension according to the ABPM measurements. The mean left ventricular mass (LVM) index was 37.5 ± 11.2 g/m^2.7^; 6 patients (27%) had LVH as LVM index >38 g/m^2.7^. The mean carotid IMT was 0.46 ± 0.06 mm, carotid IMT-SDS was 2.03 ± 1.68. Twelve patients (54%) showed increased carotid IMT. Patients had higher aortic PWV (5.32 ± 0.87 m/s) compared to both control groups (4.98 ± 0.45 m/s for control A, and 4.84 ± 0.38 m/s for control H); however the difference was significant only for control H (p= 0.041). Aortic PWV was found to be correlated with average index SBP on ABPM (r=0.74, p< 0.001) and BMI-SDS (r=0.41, p=0.036). No correlation was found between PWV and laboratory parameters including s-CRP, ESR, C_3_, C_4_, anti-ds DNA, ACP IgM or ACP IgG levels. PWV showed a significant association with carotid distensibility calculated from carotid end-diastolic and peak systolic internal dimensions from the M-mode images of carotid artery (r=-0.57, p=0.007). Aortic augmented pressure and augmentation index (AI) were similar in patients and controls.

## Conclusion

Patients with juvenile onset SLE, even without organ damage (SLICC=0), had an evidence of early atherosclerosis and increased left ventricular mass. Our result suggested that subclinical cardiovascular disease begins in the early years of life. We conclude that early identification of subclinical cardiovascular abnormalities is important to reduce overt cardiovascular disease in patients with SLE.

